# Able-Bodied Wild Chimpanzees Imitate a Motor Procedure Used by a Disabled Individual to Overcome Handicap

**DOI:** 10.1371/journal.pone.0011959

**Published:** 2010-08-05

**Authors:** Catherine Hobaiter, Richard W. Byrne

**Affiliations:** Centre for Social Learning and Cognitive Evolution and Scottish Primate Research Group, School of Psychology, University of St Andrews, St Andrews, United Kingdom; Università di Parma, Italy

## Abstract

Chimpanzee culture has generated intense recent interest, fueled by the technical complexity of chimpanzee tool-using traditions; yet it is seriously doubted whether chimpanzees are able to learn motor procedures by imitation under natural conditions. Here we take advantage of an unusual chimpanzee population as a ‘natural experiment’ to identify evidence for imitative learning of this kind in wild chimpanzees. The Sonso chimpanzee community has suffered from high levels of snare injury and now has several manually disabled members. Adult male Tinka, with near-total paralysis of both hands, compensates inability to scratch his back manually by employing a distinctive technique of holding a growing liana taut while making side-to-side body movements against it. We found that seven able-bodied young chimpanzees also used this ‘liana-scratch’ technique, although they had no need to. The distribution of the liana-scratch technique was statistically associated with individuals' range overlap with Tinka and the extent of time they spent in parties with him, confirming that the technique is acquired by social learning. The motivation for able-bodied chimpanzees copying his variant is unknown, but the fact that they do is evidence that the imitative learning of motor procedures from others is a natural trait of wild chimpanzees.

## Introduction

In recent years, a growing range of animal species has been reported to show local differences in behavioural traits that appear to be based on cultural transmission (e.g. great apes [1], [2], [3], [4], [5], monkeys [6], [7], [8], whales and dolphins [9], [10], rats [11], coral reef fish [12]). Whereas it used to be considered—because pedagogy and imitation were thought absent in non-humans and essential for culture [13], [14], [15]—that ‘animal culture’ was an impossibility, cultural traditions in animals are now accepted [16]. Indeed, their existence is now taken for granted in a range of investigations: on the ecological conditions that promote culture [17], [18]; on the extent to which animal culture is dependent on conformity bias [19]; on the kinds of information that can be transmitted culturally [20], and so forth. And, strikingly, in transmission-chain experiments with human adults, opportunities for pedagogy or imitation have been found to be no more effective than simply seeing the end products in allowing cumulative development of traditions [21].

The powerful mechanisms of social learning available to humans are evidently *not* necessary for some sorts of culture to be established. The strong sense of imitation, learning a novel procedure from seeing it done, remains controversial in animals and clear experimental evidence of it is lacking even in chimpanzees [22], [23], [24]; teaching has been clearly demonstrated in only a few species, not particularly those noted for culture [25], [26]. Does this mean, then, that the earlier insistence on the importance for animal culture of imitation and pedagogy—‘sophisticated’ mechanisms of social learning, as so-called —was simply misguided? We suggest that would be an oversimplification.

The intense interest and heated debate about animal culture [12], [20], [27], [28], [29], [30] has centred on the claims of culture *in the great apes*: for good reason [31], [32]. It is only in the great apes that there is strong evidence of organizational complexity in what are apparently learned traditions (chimpanzee: in tool-use [33], [34], [35] and with plant foods [36]; orangutan: in tool-use [37], [38] and with plant foods [39]; gorilla: with plant foods [40], [41], [42]). (But note that capuchin monkeys in an arid area of Brasil have recently been found to use tools in several ways: their behaviour shows careful selection of tools and remarkable efficiency in nut-cracking, with human-like hefting of heavy stones, so further study may reveal ape-like organizational complexity in their behaviour also [43], [44], [45], [46].) If these ape skills are indeed cultural products—and at present there is little evidence of how they are learned [47], [48]—then apes must possess sophisticated mechanisms of social learning, capable of passing on a procedural organization of actions. Thus, whether imitative learning of novel motor procedures under natural conditions is within the capacity of primates other than humans has been a topic of intense recent interest and debate [24], [32].

In this study we take advantage of a naturally-occurring situation that presents an unusual opportunity for the identification of imitation in the wild. The major impediment to detecting imitation under natural conditions is that, generally, it is not possible to identify the original model that has been copied. If the consequence of what appears successful copying is simply part of the normal behaviour for the species, it remains possible that this would have developed without any social learning. A particularly clear hallmark of human imitation is the copying of behaviour that has no useful function for the imitator: as when right-handed children taught by a left-handed teacher acquire the ‘hooked’ writing position. In a similar way, we have been able to study the copying of an unnecessary behavioural trait by wild chimpanzees, when the only original model is an individual for whom it is highly functional.

**Figure 1:**
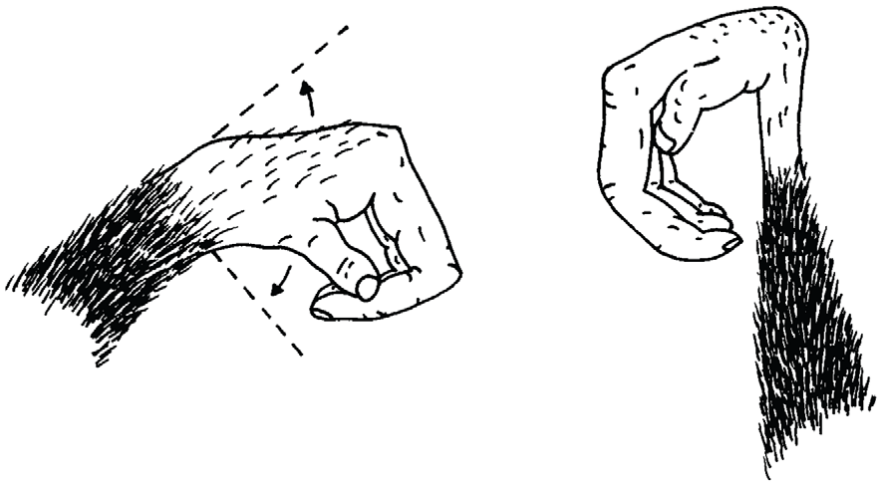
Hand injuries of male chimpanzee, Tinka. He suffers from near complete bi-manual paralysis: the fingers of both hands are permanently flexed, and both wrists are effectively paralysed.

**Figure 2:**
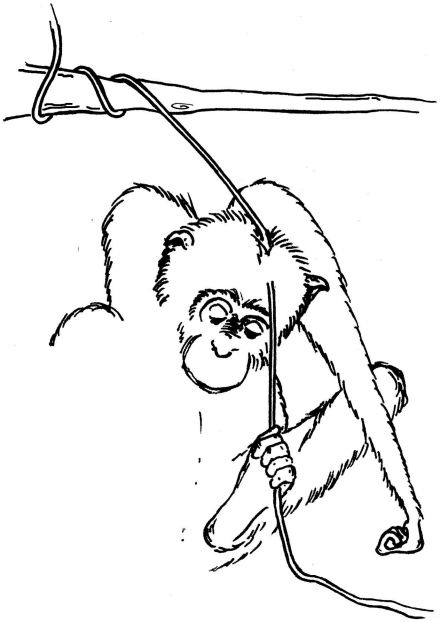
Tinka's liana-scratch technique. He uses his foot to grip and pull the liana downwards and outwards, before rubbing his head against the taught surface.

Until recently, chimpanzees of the Sonso community, Budongo, Uganda, encountered large numbers of snares intended for duiker and bush-pigs; early efforts by a four-man team to clear the area led to the removal of up to 200 snares per month [49]. The result now is that one in three adult individuals has permanent snare-related disabilities [50], and several individuals show idiosyncratic behavioural strategies that compensate their disabilities [51]. Adult male Tinka suffers from near total paralysis of both hands (Fig. 1; and see Method), precluding most normal body-maintenance by self-grooming or scratching. Tinka also suffers from a chronic skin complaint and receives low levels of social grooming. Apparently in consequence, he has developed an efficient but highly idiosyncratic alternative: liana-scratching (see supporting information: Videos S1, S2, S3 and S4, captions in Text S2). Tinka's liana-scratch technique consists essentially of grasping a growing liana, pulling it downwards or sideways in order to hold the flexible stem taut, and then rubbing his body back-and-forth against the taut liana (see Fig. 2). Imagine using a towel on one's back, except that in this case, rather than the towel moving, the liana is held taut and the body moved relative to it. Presumably because Tinka has effectively no voluntary control of his fingers, he uses his toes for the grasping and pulling; at times he increases the tension in the liana with a pull from the other foot; sometimes he uses the back of a hand or foot to manoeuvre the liana before tensioning it by grasping and pulling with a foot.

Tinka's behaviour offers regular demonstration, to any other chimpanzees that are nearby, of an organized sequence of action that is necessary for him but would not be for them: a ‘natural experiment’ that mimics the case of the child learning to write from a left-hander. All the elements of action that comprise liana-scratching are present in the normal behavioural repertoire of the Sonso chimpanzees: pushing and pulling objects, including lianas, and rubbing the body against stationary objects such as logs. However, the highly specific procedure of the liana-scratch technique has not previously been reported in any other individual at Sonso; it is absent in the detailed glossaries of chimpanzee behaviour published from Mahale [52] and Gombe [53], and from a recent extensive survey of geographic variation in chimpanzee behaviour [54]. We take it, therefore, that liana-scratch is not simply a normal but low-frequency element in the chimpanzee repertoire, but a process originated by Tinka. Here we report evidence that this disability-specific process has been imitated by able-bodied chimpanzees.

## Methods

### Study site and subjects

The Budongo Conservation Field Station (BCFS) was established in 1990 in the Budongo Forest Reserve, which lies in the western Rift Valley in Uganda (1°35′–1°55′N, 31°18′–31°42′E) at a mean altitude of 1050 m. The 793 km^2^ Reserve includes 482 km^2^ of continuous medium altitude semi-deciduous forest cover [55]. The forest within this site is, as a result of regular logging until 1990, predominantly secondary forest growth, which frequently restricts ground visibility to less than 6 m. At the start of data collection in October 2007, the Sonso study community of chimpanzees consisted of 81 named individuals. Fourteen individuals (3 juveniles and 11 adults) had permanent snare related manual disabilities. Of these, Tinka, an adult male (49±3 yrs), was the most severely injured.

Both Tinka's left and the right hand exhibit severe deformities [51]. Most of the muscles of the left wrist are apparently paralysed, which allows the left hand a limited axis of movement, but in its relaxed posture the wrist is hooked and weakened. Digits 1–4 are permanently flexed and incapable of assuming any independent movement although the thumb has retained some function. The right hand exhibits even greater deformity, with complete paralysis of the wrist and voluntary movement impossible. In addition to his injuries he suffers from a chronic skin infection that causes extensive dry, flaky skin, rash and hair loss; these symptoms are consistent with skin mite dermatitis and allergy. This appears to cause him frequent discomfort, exacerbated by the fact that the extremely limited range of movement in his wrists and fingers prevents him from using them to groom or scratch his body in a normal fashion. While he occasionally uses the side of a hand in combination with his lips to groom areas on his chest and arms, this technique is cumbersome; he is unable to groom his head, back or lower body. Here, where any normal chimpanzee would simply use a combination of scratching and grooming with both hands, Tinka uses the liana-scratch technique.

### Procedure

Observations of liana-scratch behaviour were recorded on an ad hoc basis during data collection for a project on chimpanzee gestural communication (see supporting information: Text S1). All examples of behaviour where a liana was noticed being used during a self-grooming bout were recorded on miniDV tape using a Sony Handycam (DCR–HC-55).

In addition, field assistants regularly record party composition, ranging, and the frequency and duration of behaviours such as grooming, onto handheld Workabout Pro computers [56]. All adult and independent sub-adult individuals at Sonso are scored individually. For our purposes, this means that we can track the ranging of the juveniles whose behaviour we analyse, because in this population juveniles, including individuals up to 13 years old, travel consistently with their mothers during the whole day.

### Analysis

Digital videotapes were transferred to an Apple MacbookPro computer; these were edited into discrete clips using iMovie and labelled for analysis and categorisation. Analyses were carried out in SPSS v11, with α = 0.05 required for significance. Means are given with ± Standard Deviation, throughout.

The critical decision in coding this video material was whether an able-bodied chimpanzee was using Tinka's liana-scratch technique. Assessing inter-observer reliability was not straightforward, as liana-scratch is a relatively rare behaviour: if clips of behaviour apparently matching this pattern were interspersed among randomly selected clips, then 100% inter-observer agreement would be obtained, trivially. Instead, we used a sample of video clips from able-bodied chimpanzees that contained all possible cases of liana scratch, including the 21 positive exemplars as identified by the primary coder (CH) and several other similar-looking action sequences which had been rejected as exemplars. This meant that positive cases of liana-scratch were in the majority, according to the primary coder; however, that fact was unknown to the second coder, who was made familiar with the appearance of liana-scratch from video material of Tinka's behaviour, only. Inter-observer agreement in coding material from able-bodied chimpanzees was ‘very good’, with a Cohen's Kappa of 0.85. We also investigated inter-observer reliability for the secondary decision as to how many elements of liana-scratch (of a possible three: grip liana, pull tight, rub body side-to-side) were present in each exemplar identified. Here again the agreement between coders was very good, with Kappa 0.83.

## Results

Between October 2007 and August 2009 we recorded video evidence of 21 bouts of liana-scratch (L-S), within self-grooming episodes by 7 able-bodied individuals (see supporting information: Videos S5, S6, S7 and S8). All the 7 individuals were healthy and able-bodied, and all were in the 4–13 years age range: Night (5 yr female, first showed L-S when 4 yr; 4 bouts), Zak (6 yr male; 5 bouts), Karo (7 yr female; 3 bouts), Kumi (8 yr female; 5 bouts), Zed (8 yr male; 2 bouts), Kana (10 yr female; 1 bout), Bahati (13 yr female, showed L-S when 12 yr; 1 bout). None of the bouts occurred within the same party of chimpanzees on the same day. However, video S5 shows juvenile Zed using the L-S technique just after he watched Tinka employing it, as shown in video S1. This was the second observation on which Zed was observed using the L-S technique. On no other occasion was Tinka present within the party when L-S was recorded in an able-bodied individual.

Eighteen of the recorded instances of liana-scratch by able-bodied chimpanzees could be seen clearly on the video; three were partially obscured. Of the 18 clearly visible cases, in 13—involving 6 different individuals—the technique closely mirrored Tinka's: grip liana, tension by pulling, and rub body part side-to-side. Unlike Tinka, however, able-bodied individuals normally used a hand rather than a foot to produce tension in the liana. (See Table 1 for details of variation in L-S technique among able-bodied chimpanzees.) In the remaining 5 cases, tension in the liana was attained by pushing against it with the back of the hand or wrist, rather than gripping and pulling the liana. Tinka was also sometimes noted to use pushing with back of hand or wrist, for initially manoeuvring a liana into position; however, he always used his foot to apply tension. The back-and-forth sawing motion of scratching the body against the liana was seen in every case.

**Table 1:** Actions used in L-S by able-bodied chimpanzees

Individual	Liana-scratch technique				
	Tension by pulling		Tension by pushing		Unclear
	Hand	Foot	Hand	Foot	
Bahati (13 yr. F)	1				
Kana (10 yr. F)					1
Karo (7 yr. F)		3*			
Kumi (8 yr. F)	2		3		
Night (5 yr. F)	2		2		
Zak (6 yr. M)	4				1
Zed (8 yr. M)	1				1
	**10**	**3**	**5**		**3**

*In these 3 cases Karo added a second grip with the hand so that both hand and foot pulled on the climber

We used long-term project records [56] to investigate the opportunities, available to able-bodied individuals showing liana-scratch, for learning from Tinka's behaviour. The range of the Budongo chimpanzees is conventionally divided into the ‘core’ area and the ‘periphery’; Tinka's home range lies entirely in the core area. We examined all able-bodied chimpanzees in the 4–13 year age range for whether their range overlapped that of Tinka or not (i.e. whether their mother's range was core or peripheral, since all these individuals were reliably found with their mother). As we were aware of a potential bias towards the observation of core individuals, we tested the number of individuals in which liana-scratch was observed, rather than the number of cases of liana-scratch. To ensure that all individuals showing liana-scratch had been identified, we interrogated all other researchers and field assistants working at Budongo. Use of the liana-scratch technique was significantly associated with sharing the range area of Tinka (Yates' corrected Chi-square test, one-tailed: among 4–13 yr immatures, n = 19, χ^2^ = 4.20, df = 1, p = 0.02).

Individuals might share the same range, yet not associate with each other in the same foraging parties, and thus lack real chances to observe others' behaviour. That was not the case for the individuals showing liana-scratch. During the year 2008, the able-bodied chimpanzees that showed liana-scratch were recorded in a group with Tinka during more than twice as many hours as those in whom the behaviour was absent (mother's time with TK: for all mothers of individuals in whom L-S present: n = 6, mean  = 194.8±48.1 hrs: for all mothers of individuals in whom L-S absent: n = 8, mean = 78.3±28.3 hrs; t-test: t = 5.71, df = 12, p = 0.01).

## Discussion

The ‘natural experiment’ of the presence of disabled individuals in this chimpanzee population has allowed behavioural strategies to develop which can be clearly differentiated from the natural repertoire of an able-bodied chimpanzee, for whom they have no apparent function. Moreover, the disability-specific nature of some of these strategies allows particular individuals to be pinpointed as the only possible models for copying: specifically, in the case of liana-scratch, Tinka. The absence of liana-scratch in previous observations, at this or any other long-term chimpanzee site, implies that liana-scratch is an innovation by the disabled chimpanzee Tinka, for whom it is highly functional. Tinka's skin complaint, lack of regular grooming by others, and severe bi-manual disability mean that the liana-scratch technique allows him considerable gains in skin-care and consequent comfort; and the actions which are coordinated together to produce the novel pattern are ones that even the disabled Tinka can do. Body maintenance by liana-scratch does not appear to offer any benefit to able-bodied chimpanzees, however, since they are able to scratch themselves, self-groom, and solicit grooming from others.

Nevertheless, we found liana-scratch to be used by several able-bodied individuals as well. All the able-bodied chimpanzees to use liana-scratch were resident in the same area as Tinka, whereas none of the chimpanzees that did not share Tinka's range has ever been seen to use this idiosyncratic technique. Moreover, those chimpanzees that used the technique were much more often actually present in parties with Tinka than similar aged chimpanzees that did not. (An able-bodied chimpanzee might, of course, have learnt liana-scratch at one remove, from another able-bodied chimpanzee already using the technique; but we have no evidence that this occurred.) We therefore conclude that observation of an individual who shows liana-scratch is necessary and may be sufficient for chimpanzees to learn this novel behaviour pattern.

Observations of able-bodied chimpanzees using the liana-scratch technique were not clumped into a few episodes, where one individual's behaviour might have been facilitated by seeing another's, but rather each case was noted on a different day or in a different chimpanzee party. However, on one occasion, a juvenile who had just watched Tinka use the technique then used it himself shortly afterwards (see videos S1 and S5). Tinka's disability is longstanding, so his compensatory liana-scratch technique is unlikely to be recently developed; it is therefore interesting that the only able-bodied chimpanzees to use liana-scratch were all young individuals. Previous cohorts of young chimpanzees may also have copied liana-scratch, but abandoned it in the face of the greater ecological demands of adulthood when they found it offered no benefit to them. Why able-bodied chimpanzees should copy this technique, we do not know. However, behavioural ‘fads’ have previously been recorded in captive chimpanzee groups [57], and in one case several juveniles apparently mimicked the strangely hunched style of walking of one older individual [58]. Our observations suggest that such fads and mimicry, although biologically functionless where they have been noticed, reflect a natural trait of wild chimpanzees that may be an important component of the cultural transmission of valuable survival skills.

The fact that liana-scratch was acquired only by those able-bodied young chimpanzees that had ample opportunity to observe Tinka's unique technique of self-scratching, and was employed even when not with the original model, allows clear documentation of the chimpanzees’ ability to acquire a novel motor procedure by social learning. No teaching was involved, and simple mechanisms such as stimulus enhancement and response facilitation (though likely involved) would not be sufficient to allow replication of behavioural organization [22], [23], [59]. In stimulus enhancement [60], seeing a conspecific at a place or interacting with an object increases the probability of oneself subsequently interacting with those things; in response facilitation [61], seeing a conspecific executing an action that is also in one's own repertoire increases the probability of subsequently activating that action. These phenomena can be understood as ‘priming’ of pre-existing brain records corresponding to objects or actions [24], but they cannot account for acquisition of a novel procedure We consider, therefore, that some sort of imitation is implied: but which, of the several mechanisms that have been proposed?

Experimental studies have explored the imitative abilities of chimpanzees [62], [63], [64], [65]. In these cases, the actions are ones the subjects can already do: what is learned is not a new action, but the appropriate circumstances in which to deploy a familiar one, a process termed contextual imitation [23], [59]. The learning is a matter of selection from, not extension to, the existing repertoire; and this may be based on relatively simple cognitive mechanisms [22], [23], [59], [61], [66]. Even gestural imitation [67], in which apes are trained to ‘do as I do’ and then presented with seemingly novel actions to copy, may reflect the same process of selection rather than learning of new procedures. Unlike most animals, apes have very large repertoires in which many of the actions are latent or seldom used [68]. In the only study of great ape gestural imitation in which the subject's repertoire could be traced back over many years, all the ‘imitations’ of seemingly novel actions proved to have been made before [69]. Although they resembled the demonstrated action closely enough to be reliably identified by naïve coders, the match was sometimes inexact, as is found with all such studies—to be expected, if the ‘copies’ were selected by resemblance from the existing repertoire. However, contextual imitation, in which pre-existing behavioural routines are selected on the basis of physical match by observation of another's behaviour, is not sufficient to explain copying of liana-scratch, an organized, goal-directed sequence of actions that does not normally occur in the chimpanzee repertoire: procedural imitation is required.

A distinction, introduced by the developmental psychologist Wood [70], has been found helpful in categorizing two kinds of procedural imitation: impersonation and emulation [71]. In *impersonation*, sometimes described as “true imitation”, the imitator tries to behave as like the model as possible: the result is a close match in specific details of behaviour. In the case of able-bodied chimpanzees acquiring liana-scratch, that was clearly not the case. Easily-observed details of how tension was applied to the liana were not copied: Tinka always gripped the liana with his toes, whereas able-bodied chimpanzees gripped or pushed the liana with a hand. In *emulation*, learning proceeds by means of copying end results rather than actions. Evidence of learning new motor skills by imitation has often been ambiguous in chimpanzees and other great apes: in many cases that superficially suggest impersonation the evidence is equally consistent with emulation [71]. Indeed, the ability to impersonate has sometimes been argued to be uniquely human [15], [72], [73]; emulation has therefore been considered primitive compared to impersonation, although in child development the ability to emulate appears much later [74]. The “result” of liana-scratch is evidently body-maintenance, so emulation learning seems prima facie irrelevant. However, the scope of emulation learning can be broadened to include observational learning of the “affordances” of objects and actions [71]: physical properties and cause-and-effect relationships. In the case of liana-scratch, affordances might include the fact that pushing/pulling on a liana makes it rigid, more like a growing tree-trunk that the body can be rubbed against. A case might therefore be made that the able-bodied chimpanzees learnt this affordance from watching Tinka's actions, and thus discovered an efficient method of body maintenance that they would not otherwise have worked out. However, this particular affordance seems unlikely to need the help of an animate demonstrator, and would be much more apparent in personal exploration. Young chimpanzees are active and investigative, and in the wild spend substantial periods playing with and climbing upon lianas, during which the effect of tension is repeatedly made evident. Moreover, there is no reason to suppose that liana-scratch is particularly efficient in body-maintenance for a chimpanzee that can simply scratch with a hand or solicit grooming from another chimpanzee. Instead, the fact that only juvenile chimpanzees show liana-scratch, and then apparently give it up when they become adult, implies that a tendency to imitate rather than discovery of an affordance is the basis of the phenomenon.

Imitation of the overall organization of motor actions, without necessarily duplicating (as in impersonation) the precise actions, has been defined as *program-level imitation* [59], [75]. In program-level imitation, the logical, hierarchical organization—the “gist” of a behavioural routine—is copied, by putting together a novel organization of pre-existing components of the imitator's behaviour repertoire. The precise details of actions may not be copied, since each step in the overall process is achieved using an action familiar to the imitator. This makes program-level imitation highly efficient: if the imitator is an infant, and the model much larger and stronger, precise copying would be likely to fail. An example from human development is the imitation of new words by a young child. Characteristic mispronunciations and shifts in vowel pitch, between adult model and child's copy, betray the fact that the child parses the word into phonemes, learns only their sequential organization by imitation, and utters a copy that is made up of her own motor programs for generating phonemes. Program-level imitation has been argued to underlie great apes' learning of novel feeding routines, because it does not require a rich understanding of intentions and causality [40], [76]. Instead, the logical structure to be copied is parsed from observing the behaviour repeatedly; such parsing only requires detection of the statistical regularities underlying the efficient use of coordinated actions [77], [78]. Program-level imitation is fully capable of explaining the copying of liana-scratch by able-bodied Sonso chimpanzees.

We conclude that the cognitive capacities, underlying the spread of liana-scratch to able-bodied young chimpanzees, are (1) the chimpanzee's ability to copy an organized procedure composed from several simpler actions already within the normal species repertoire, most simply characterised as program-level imitation, and (2) the chimpanzee's natural tendency to copy novel organized, goal-directed actions that it repeatedly sees demonstrated by others. These traits are directly relevant to the question of how the elaborate technical skills of the chimpanzee behaviour are acquired socially: chimpanzees are able to learn novel behavioural routines by imitation.

## Supporting Information

Text S1 Details of chimpanzee injuries, chimpanzee ranges, and methods used to sample individuals for study.
(0.05 MB DOC)

Text S2 Descriptions to accompany video clips.
(0.02 MB DOC)

Video S1 Tinka R-side. Liana scratch of right side, by Tinka.
(5.25 MB MOV)

Video S2 Tinka L-side. Liana scratch of left side, by Tinka.
(4.83 MB MOV)

Video S3 Tinka arm. Liana scratch of arm, by Tinka.
(3.67 MB MOV)

Video S4 Tinka head. Liana scratch of head, by Tinka.
(3.43 MB MOV)

Video S5 Zed. Liana scratch by zed.
(1.49 MB MOV)

Video S6 Karo. Liana scratch by Karo.
(3.29 MB MOV)

Video S7 Night. Liana scratch by Night.
(2.20 MB MOV)

Video S8 Night. Liana scratch by Night.
(1.76 MB MOV)
