# Supplementary material for: Able-Bodied Wild Chimpanzees Imitate a Motor Procedure Used by a Disabled Individual to Overcome Handicap
Source: PLoS One. 2010 Aug 5;5(8):e11959. doi: 10.1371/journal.pone.0011959 (PMC2916821; doi:10.1371/journal.pone.0011959)
Supplement: Text S1 — Details of chimpanzee injuries, chimpanzee ranges, and methods used to sample individuals for study. (0.05 MB DOC) [file pone.0011959.s001.doc]

Supplemental information

*Snare injuries*

A large number of the Sonso chimpanzees suffer from injuries caused by snare traps, left in the forest by bush-meat hunters from local villages. Commonly fashioned from wire or nylon, they trap the limbs of passing chimpanzees and are pulled tight across the wrist or ankle as the chimpanzees struggle to remove them. In the case of nylon snares they can be chewed off before permanent damage is caused; however, wire-snares can remain in place for weeks or months cutting circulation and causing severe nerve and tissue damage. In the long term this can result in permanent paralysis, wastage and occasionally amputation.

*Core and peripheral chimpanzees*

Since the subjects of this paper were immature chimpanzees, all still ranging with their mothers, we used the mother’s ranges as a proxy for theirs. Female chimpanzees have a smaller range than males, within the male chimpanzee community territory [1]. Sonso females are divided into core individuals, who spend the majority of their time within the central area of the community territory, and peripheral females, who spend the majority of their time in areas bordering (and sometimes overlapping) the territory of other communities. Of the 14 regularly seen parous females, 7 are core and 7 peripheral, including core female Zana, mother of the brothers Zalu and Zed. Her death in 2007 orphaned these immatures, but they have retained core ranges.

Sonso males spend the majority of their time in the central core of the community range [2]; consequently, core females and their dependent offspring spend more social time with males than do peripheral females and their offspring. The Sonso community home range is approximately 7 km^2^; however, adult males with snare injuries have smaller individual ranges, probably as a result of the increased travel costs resulting from their injuries. Tinka’s range was identified in 2003 as the smallest, with an estimate of only 1-3km^2^ [2, 3].

*Sampling procedure*

Observations were made on all chimpanzees within the Sonso community during three field periods between October 2007 and August 2009 (Oct 07-Mar 08; Jun 08-Jan 09; May 09-Aug 09; total 266 days of observation time). Primary data were recorded on an *ad libitum* basis between 7.30am and 4.30pm, on a schedule of 3 days on, 1 day off, 3 days on, 2 days off. Because researchers of the Budongo Forest Project follow chimpanzee individuals of the habituated group daily, locating chimpanzees did not normally present difficulties; however, location of specific individuals could be problematic. The combination of low visibility in a dense rainforest environment and varied levels of habituation among individuals also limited opportunities to capture clear video footage of social interactions. We therefore used a *focal behaviour* sampling approach {Altmann, 1974 #237}, and maintained a record of the frequency with which a particular individual was observed; where we had to choose which of several social interactions to film, we targeted individuals previously sampled infrequently. Sampling was conducted by CH during an ongoing study of gestural communication; the observations, although not made ‘blind’, were video-recorded simply as cases of apparently interesting behaviour, with no prior idea of demonstrating imitation in a wild population of chimpanzees.

Supplemental References

1. Wrangham, R.W. (1979). Sex differences in chimpanzee dispersion. In The great apes, D.A. Hamburg and E. McCown, eds. (Menlo Park CA: Addison-Wesley), pp. 481-489.

2. Newton-Fisher, N.E. (2003). The home range of the Sonso community of chimpanzees from the Budongo Forest, Uganda. African Journal of Ecology *41*, 150-156.

3. Newton-Fisher, N. (2000). Male chimpanzee core areas: ranging in Budongo Forest chimpanzees. Pan African News *7*, 10-12.
