# Supplementary material for: Able-Bodied Wild Chimpanzees Imitate a Motor Procedure Used by a Disabled Individual to Overcome Handicap
Source: PLoS One. 2010 Aug 5;5(8):e11959. doi: 10.1371/journal.pone.0011959 (PMC2916821; doi:10.1371/journal.pone.0011959)
Supplement: Text S2 — Descriptions to accompany video clips. (0.02 MB DOC) [file pone.0011959.s002.doc]

Video S1: Tinka-RSide

(All four clips of Tinka’s liana-scratch technique are taken from the same occasion.) Tinka is in the centre of the frame, flanked by Zalu on the left and Zed on the right. Tinka grabs and pulls the liana taught with his right foot, then increases the tension with a second grab and pull from his left foot before starting the rubbing motion at his right shoulder. As well as the back-and-forth rubbing motion, he also pushes his body forwards, rolling the climber along the side of his body and so moving the location of the scratching. Once he has finished a complete scratch from shoulder to hip, he moves the liana back to his shoulder and repeats the process.

Note that as Tinka starts to use the liana-scratch technique, the immature individual on the left (Zed) turns his head to watch closely. Zed later moves to the same climber and starts to initiate a liana-scratch (shown in Video 5: LS-Zed).

Video S2: Tinka-LSide

After completing a liana-scratch to the left side of his body, Tinka (centre-frame) moves the liana over to the right side of his body and repeats the procedure, gripping and pulling the liana taught with his right foot. Tinka’s manual disabilities are clearly displayed at the start of the clip as he struggles to manoeuvre the liana across to the new position. Zed is still watching carefully on the left hand side of the screen.

Video S3: Tinka-Arm

Tinka (centre-frame) grabs and pulls the liana taught with his right foot; he then rubs the outside and inside of his right arm back and forth against the liana. Note that after the liana-scratch he closely peers at his arm possibly searching for any parasites that may have been dislodged.

Video S4: Tinka-Head

Tinka (centre-frame) grabs and pulls the liana taught with his right foot, holding it in position in front of him and rubbing his head back and forth against the taught liana.

Video S5: Zed

Immediately after Tinka moves away from the liana he had been using for liana-scratch (see videos 1-4), young male Zed, who had been observing his liana-scratching closely, moves towards the liana. He grabs and pulls it semi-taught with his right hand and starts to rub the back of his left forearm against it. However, his older brother Zalu (seen crossing screen from left) moves away and Zed stops and later follows him. He was also seen to produce the complete liana-scratch on other occasions.

Video S6: Karo

Young female Karo grabs the liana with both her right hand and her left foot and then pulls the liana taught with her right hand. She then rubs the back of her left forearm back and forth against the liana, before moving the liana around her elbow and repeating the back and forth rubbing motion in the new location on her upper arm.

Video S7: Night

Young female Night has grabbed the liana with her left hand and pulled it down and taught. She then rubs the underside of her chin back and forth against it.

Video S8: Night2

Young female Night uses her left forearm to push the liana out and keep it taught, then rubs the left side of her head and neck back and forth against it.
